# Airborne Particulate Matter Sensing via Laser Filament–Interaction and Deep Learning

**DOI:** 10.1021/acsestair.5c00362

**Published:** 2026-01-29

**Authors:** James A. Grant-Jacob, Ben Mills

**Affiliations:** Optoelectronics Research Centre, 7423 University of Southampton, Southampton, SO17 1BJ, United Kingdom

**Keywords:** particulates, remote sensing, airborne, filamentation, scattering, lasers

## Abstract

Airborne particulate matter, including mineral dust and biological aerosols, such as pollen, presents growing challenges to public health, agriculture, and environmental monitoring. Current technologies lack the capability to identify single particles remotely in real-time. Here, we demonstrate the feasibility of femtosecond laser filamentation combined with optical imaging and lightweight deep learning for the remote identification of airborne particulates. Chalk dust, pollen grains, and salt crystals were delivered into a laser-generated filament, where their optical emission events were captured and analyzed. A convolutional neural network trained on these events achieved high classification accuracy across all categories, with a mean accuracy of 87.5%. Grad-CAM visualizations confirmed that the network focused on discriminative spatial and spectral features for chalk, salt, and pollen. This study demonstrates the feasibility of remote, species-level airborne particulate detection and lays the foundation for intelligent, real-time atmospheric sensing platforms.

## Graphical abstract

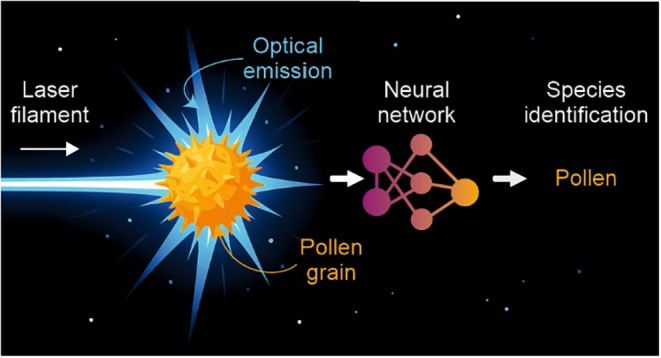

## Introduction

Airborne particulate pollution is a global health crisis,^1–5^ responsible for millions of premature deaths each year and is linked to respiratory diseases, cardiovascular issues, and reduced life expectancy.^6,7^ Yet, current methods for monitoring airborne particulate matter, especially at long-range and in outdoor, variable environments, remain limited in both coverage and specificity. Conventional systems typically rely on filter-based sampling followed by offline analysis^8–10^ or utilize optical measurements^11,12^ that generally cannot resolve individual particle types or sizes.^12–14^ These limitations hinder efforts to provide timely environmental assessments, issue health alerts, and track the movement of biologically or chemically relevant aerosol particles.

Remote sensing technologies such as LiDAR (light detection and ranging) offer powerful tools for probing objects at a distance.^15^ However, most LiDAR systems provide only bulk backscatter intensity and are not optimized for species-level discrimination.^16,17^ While developments in multiwavelength and depolarization-based LiDAR have improved aerosol classification,^18^ the technique struggles to resolve small, sparsely distributed particles or distinguish between biological and nonbiological content. In contrast, laser filamentation, which is the formation of a long, thin channel of light and plasma by an intense, ultrashort laser pulse,^19–23^ offers new ways to probe faraway objects. It has emerged as a promising tool for atmospheric diagnostics,^24^ capable of generating strong backscattered signals including broadband supercontinuum, fluorescence, and plasma emission from gases and aerosols. These filaments can propagate over several meters to kilometers in air,^25,26^ forming self-guided plasma channels with high peak intensity, making them ideal candidates for remote, real-time optical interrogation.

Unlike prefilament plasma generation, which can be spatially limited and unstable, full filamentation provides a self-guided propagation regime with clamped intensities over several millimeters and longer. This extended high-intensity interaction volume significantly increases the likelihood of particle–light interactions, leading to more consistent signal generation and enabling reliable species-level discrimination. As such, filamentation is not merely a means to reach high peak intensities but a critical mechanism for achieving stable, real-time interrogation of single particles at a distance. Despite this promise, the use of filaments to detect and classify airborne particles remains underexplored. Existing filament-based approaches typically focus on gas detection or bulk aerosol effects without targeting individual particle events.

Several optical systems have previously demonstrated particle-type discrimination by using fluorescence- and imaging-based techniques. For example, Kaye et al.^27^ developed a low-cost multichannel aerosol fluorescence sensor for networked deployment, relying on UV excitation to detect biological particles. Similarly, Könemann et al.^28^ introduced the spectral intensity bioaerosol sensor (SIBS), which measures spectrally resolved fluorescence of single particles in real-time to enable faster and more accurate classification of bioaerosols. Automated pollen counters, such as the Rapid-E system reported by Šauliene et al.,^29^ combine imaging and fluorescence-based approaches for real-time monitoring of airborne allergens. While these systems have proven effective, they require dedicated excitation sources and close proximity to the sample. In contrast, our approach, which uses a filament-induced plasma emission for airborne excitation, offers the potential for remote sensing over tens to hundreds of meters. This proof-of-principle study characterizes particle–filament interactions in a controlled near-field configuration, forming the basis for future techniques that can exploit filament propagation for long-range aerosol detection.

In this study, we demonstrate a new approach that combines ultrafast laser filamentation with high-resolution imaging and deep learning^30^ to detect and classify airborne chalk dust, pollen grains, and salt crystals from their optical emission events upon filament interaction arising from particle ablation and fragmentation in the filament. Deep learning is a machine learning approach that has previously been applied to the classification of spectra,^31,32^ images,^33^ scattering patterns,^34^ and holographic images^35^ of airborne particulates. By training a network on labeled video frames from filament–particle interaction, we demonstrate that airborne particulates can be identified from single-shot optical emission events. Although the configuration discussed in this article presents a near-field proof-of-concept for filament-induced emission imaging and classification in a controlled environment, the approach demonstrates the potential for particle detection using a filament that has the potential to be extended over large distances, enabling remote sensing for air quality monitoring.

## Experimental Methods

### Laser Filament Generation and Optical Setup

Femtosecond laser pulses (190 fs, 1030 nm, 6 kHz repetition rate, 6 W PHAROS Light Conversion) were focused into ambient air in a Class 4 laser laboratory using a 100 mm focal length plano-convex fused silica lens. The lens position was controlled using three manual translation stages to control the position of the lens in XYZ (PT1, Thorlabs). The input beam diameter was 8.9 mm (1/e^2^), and the pulse energies were scanned from 20% to 100% of the laser’s maximum output (0.2–1.0 mJ per pulse, corresponding to diffraction-limited peak intensities of ∼1.1 × 10^14^ to ∼5.6 × 10^14^ W/cm^2^) to confirm filament formation and identify the regime in which particle emission signals were detectable while maintaining filament stability. Consequently, all particle delivery and imaging were conducted at the laser’s maximum pulse energy to ensure robust and consistent emission signals for analysis.

The optical layout is shown in Figure 1a. A long-working-distance 50× objective (Olympus SLMPLN, NA 0.35) was used to collect light emitted perpendicularly from the filament. Such a lens allowed a long working distance of 18 mm from the filament. The objective was mounted on a translation stage and aligned using a high-resolution color CMOS (complementary metal oxide semiconductor) camera (Basler daA1920-160uc, 1920 × 1200 pixels). Once aligned, the camera was detached from its magnet mount, and a fiber-coupled spectrometer (B&W Tek Glacier X, cooled CCD (charged coupled device)) was positioned in its place using an SMA-to-SM1 adapter (CVH100-COL Thorlabs) with a 25 mm plano-convex lens to guide the collimated light into the fiber. This interchangeable configuration allowed the same optical axis to be used for both alignment via imaging and spectral measurements. In addition, another high-resolution color CMOS camera (Basler daA1920-160uc, 1920 × 1200 pixels) with a 35 mm lens (Tamron) was positioned on the opposite side and used to collect an image of the whole length of the laser-induced ionization to monitor filamentation.

**Figure 1:**
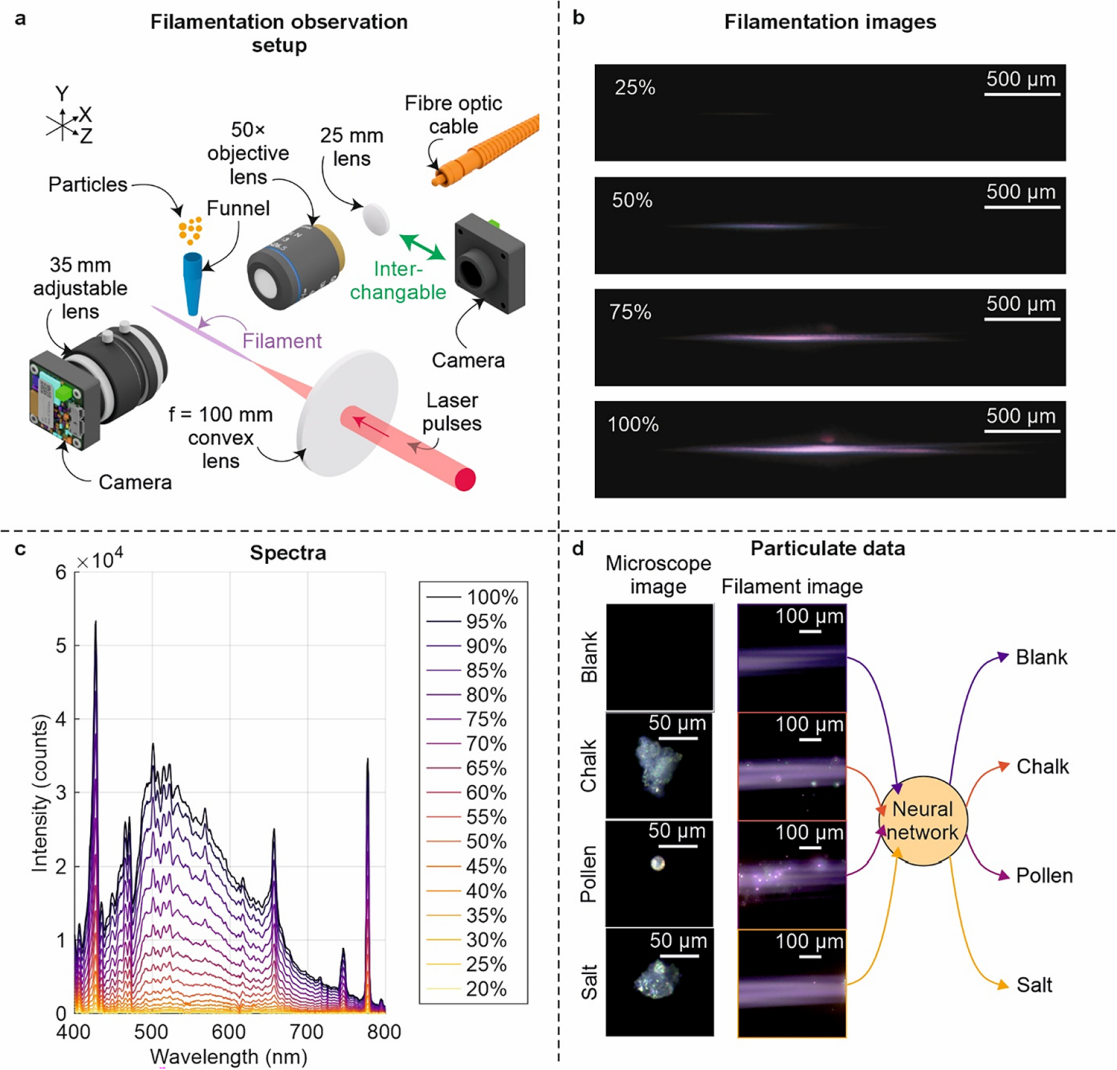
(a) Experimental setup for filament-based airborne particulate detection. Schematic showing 1030 nm 190 fs laser pulses focused using a 100 mm focal length lens into ambient air to generate a laser filament. Airborne particulates (chalk, pollen, and salt) are delivered into the beam path via a funnel placed 500 μm above the filament. Optical emission events were imaged orthogonally using a Basler RGB camera (daA1920-160uc, 1920 × 1200 pixels) coupled with a 50× Olympus long-working-distance objective (SLMPLN, 0.35 NA). (b) Plasma channel images at increasing power, showing extension, splitting, and intensity growth with increasing pulse energy. (c) Spectra for different laser powers taken for 5 s integration times showing broadening beyond 550 nm and onset of filamentation at ∼85% power. (d) Examples of microscope images of blank (i.e., nothing), chalk, pollen, and salt, along with an example of a corresponding filament image with optical emission events being fed into the neural network for prediction. Note that the brightness of the filament images has been increased for ease of viewing.

Figure 1a also shows the experimental setup used to observe filamentation, where femtosecond laser pulses are focused through an f = 100 mm convex lens and directed into the imaging system, which includes a 50× objective lens, a 25 mm lens, a fiber optic cable, and a camera. Side-view images of the plasma filament at increasing laser powers (25%, 50%, 75%, and 100%) are presented in Figure 1b. These images reveal progressive filament extension, splitting, and increased brightness with power. At full power, the filament reaches a length of approximately 3 mm, as measured using a second Basler camera, consistent with the clamped intensity regime typically associated with filamentation. Notably, filament splitting is clearly visible at 100% power, indicating strong nonlinear propagation effects.

Figure 1c shows the plasma emission spectra recorded at increasing laser powers ranging from 20% to 100%. The spectral data provide further evidence of filamentation onset and evolution. As the laser power increases, the spectra exhibit clear signs of spectral broadening, a hallmark of nonlinear optical processes such as self-phase modulation and plasma generation. Additionally, the intensity of the spectra plateaus at higher powers, indicating intensity clamping, which is a key signature of filamentation where the peak intensity stabilizes due to the dynamic balance between Kerr self-focusing and plasma defocusing. Side-view imaging shows filament splitting most clearly at 100% power, and the spectra indicate onset near ∼85%, where continuum broadening begins. Beyond this point, spectral growth without further blue-shift suggests the onset of intensity clamping. While visible plasma channel formation becomes clearly visible at approximately 75% of the maximum power, with broadband emission visible, full filamentation, which is characterized by a self-guided, high-intensity channel with stable plasma emission and spectral intensity clamping, occurs closer to 85%. This filamentation regime was selected for subsequent measurements because it provides a more intense and extended stable interaction region, ensuring reproducible ablation and scattering signatures and reducing variability.

Figure 1d presents microscopy images of representative particulates, namely, blank (the filament without added particulates used as a control), chalk, pollen, and salt, alongside corresponding optical emission events captured within the filament. It should be noted that particles entering the filament are likely ablated, partially ablated, or fragmented, and the emission signatures therefore arise from these smaller fragments or plasma rather than intact grains. These images are used as inputs to a neural network, which classifies the particule type based on learned features.

Airborne particle concentration within the laboratory was assessed using a Burkard Personal volumetric air sampler operating at 10 L min^–1^ for a duration of 9 min, corresponding to a sampled volume of 0.09 m^3^. The experiment was conducted under controlled conditions (temperature 21 °C, relative humidity 34%) with a polystyrene shielding positioned around the sampling region to minimize airflow within the filamentation region. Particles deposited on the impact surface were imaged using dark-field microscopy and analyzed through automated segmentation based on global intensity thresholding with a size inclusion criterion of ≥3 μm equivalent diameter. Across the sampled area, 342 particles were detected, yielding an estimated concentration of approximately 3.4 × 10^3^ particles m^–3^ and a surface density of 28 particles mm^–2^. The mean particle size was 6.0 μm (median, 3.9 μm). These measurements provide an indicative baseline for a shielded laboratory environment.

### Spectral Characterization

Spectra were collected at varying power levels with 5 s integration times and dark subtraction. Following this, the camera was repositioned to capture emission events during particle delivery and interaction with the filament.

### Sample Preparation

Pollen from*Iva xanthiifolia* species was obtained commercially (Sigma-Aldrich, P7395-1G), chalk dust (primarily CaCO_3_) was obtained from white chalk sticks (Crayola No. 280), and ground salt crystals (primarily NaCl) were obtained from Saxa Fine Table Salt. Particles were delivered into the beam under still-air conditions by releasing them with optical-grade cotton-tipped applicators (Thorlabs CTA10, 6″ wood stick, nonabrasive cotton tips) into a 2 cm long coned polypropylene funnel (5 mm input diameter, 2 mm exit diameter) positioned 500 μm above the laser filament path. The swabs were changed between samples, handled with sterile gloves, and the funnel was cleaned using acetone between each deposition to minimize cross-contamination.

Measurements of the particle sizes were performed by using a Nikon Eclipse microscope equipped with a 20× LU Plan ELWD 0.4 NA objective to take images (under dark-field illuminated) of the particles deposited onto a glass slide (25 × 75 × 1 mm^3^, soda lime) using the same method as for depositing through the filament beam. Image scale was calibrated using a stage micrometer (graticule), giving approximately 0.192 μm per pixel. The range of particle sizes, along with the mean and standard deviation, was 3–28 μm for chalk dust (9.4 ± 4.1 μm), 4.6 and 30.7 μm for pollen grains (20.7 ± 3.8 μm), and 12–37 μm for salt crystals (21.8 ± 6.8 μm). Sizes were determined via image processing code in MATLAB 2024a. Shape analysis revealed notable differences between classes, with chalk particles exhibiting mean circularity and eccentricity of 0.74 ± 0.08 and 0.60 ± 0.15, pollen grains 0.86 ± 0.11 and 0.55 ± 0.17, and salt crystals 0.70 ± 0.07 and 0.66 ± 0.17. These metrics indicate that pollen grains are generally more spherical, while salt and chalk show greater elongation and irregularity. Such morphological variability would influence ablation behavior and thus emission and classification complexity. Figure 2 illustrates the morphology trends, showing diameter versus circularity alongside boxplots of size variability.

**Figure 2:**
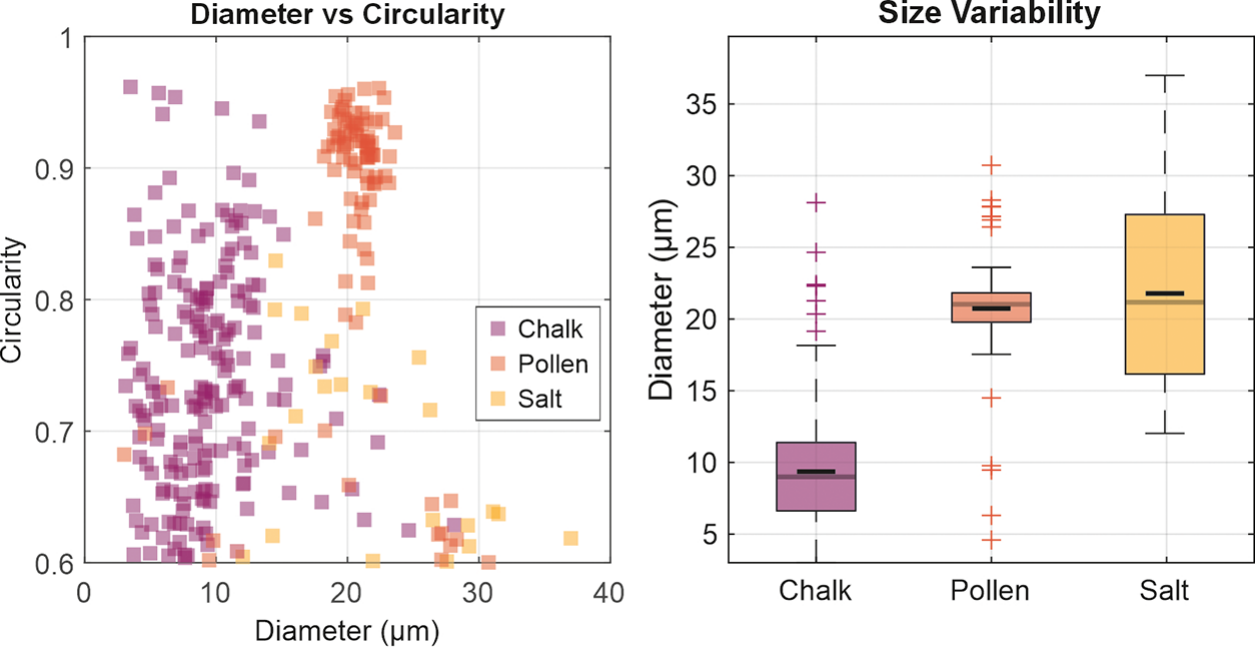
(Left) Scatter plot of diameter versus circularity for chalk, pollen, and salt particles. (Right) Boxplots showing size variability per class with mean and standard deviation annotations. Pollen exhibits higher circularity and lower eccentricity compared to chalk and salt, consistent with its more spherical morphology.

It was found that no cotton debris was present in any of the images following the deposition onto the slides. As such, it is unlikely that any cotton fibers were passed through the filament during deposition, and since the cotton swab’s fiber dimensions, when measured under the microscope, were found to be significantly larger than target particles, any fibers deposited in the beam path would have been easily distinguishable during ablation. Furthermore, because the same type of applicator was used for all samples, such events would have been consistent across the three particle classes.

By imaging the filament region with a 50× objective, we illuminated the dispersed particles using a white-light LED source (Thorlabs, PSX501) as they passed through the funnel. To avoid ablation, we captured nine images per class in the absence of the filament. Within the filament region, particle counts averaged 5 for chalk, 1 for pollen, and 3 for salt.

### Machine Learning Classification

Video frames were extracted from 10 s recording sequences at 30 fps, 50 ms integration time, and manually labeled according to known delivery species, namely, chalk, pollen, salt, and blank background. Each image was acquired at 1920 × 1200 pixel resolution and cropped into two 960 × 960 pixel segments, centered vertically to retain the filament region while removing peripheral artifacts. These cropped images were then organized into class-specific folders and used to construct a labeled image datastore. Prior to training, all images were resized to 512 × 512 pixels to match the input dimensions of the neural network. The final training data set consisted of 71 images for blank, 59 images each for chalk, 63 images each for pollen, and 33 images each for salt, totalling 226 curated samples. From this amount, 180 images were used for training and 46 for validation, with an additional 26 images placed in a separate folder for final testing and analysis. This ensured that the model was evaluated on unseen data while maintaining a reasonably balanced training set across all four classes.

A convolutional neural network (CNN) was trained in MATLAB to classify color images of the filament and any emission events. The network consisted of four convolutional blocks, each employing convolutional layers with 3 × 3 kernels and the same padding, followed by batch normalization and ReLU (rectified linear unit) activation functions.^36^ The first three blocks contained two convolutional layers, each enabling deeper feature extraction prior to downsampling. Max pooling layers with a stride of two were applied after each block to progressively reduce spatial dimensions from 512 × 512 to 256 × 256, then to 128 × 128, and finally to 64 × 64. The number of filters increased across the blocks to capture hierarchical features, specifically 32 filters in the first block, 64 filters in the second block, and 128 filters in the third. A fourth block comprising a single convolutional layer with 256 filters was included to enhance the high-level feature abstraction. To mitigate overfitting, a dropout layer with a rate of 0.5 was applied before the classification head. The classifier comprised two fully connected layers, such that the first consisted of 128 units and ReLU activation and the second mapped to the number of output classes, followed by a softmax layer and a categorical classification layer. The input layer accepted 24-bit RGB images of size 512 × 512 × 3, and the output was a single class output (either blank, chalk, pollen, or salt). A schematic of the neural network architecture is illustrated in Figure 3.

**Figure 3:**
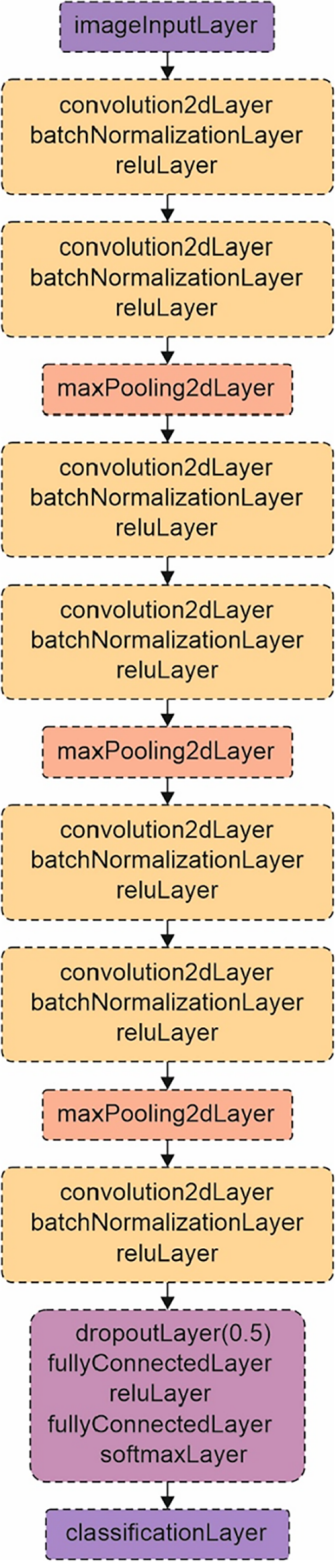
Architecture of the CNN trained and implemented in MATLAB.

Training was performed using the Adam optimizer^37^ with an initial learning rate of 1 × 10^–4^ and a piecewise schedule that reduced the learning rate by a factor of 0.1 every 30 epochs. The network was trained for 60 epochs with a minibatch size of eight, and the data were shuffled at every epoch. Validation checks were conducted every 100 iterations. While the data set size is modest and may increase overfitting risk, the applied augmentation increases effective sample diversity. Data augmentation included random X and Y image reflection, random image rotation of ±5°, random image scale change in Y and X of 0.95 to 1.05, and also included random vertical translation of the image within ±64 pixels (one-eighth of the image dimension) to improve generalization while preserving the spatial integrity of the features. Translating in X was not performed since translating any significant amount could remove important emission events. The categorical cross-entropy loss function was used for optimization.

## Results and Discussion

The results of the classification at 100% laser power are displayed in Figure 4a as a confusion matrix, where a higher percentage and brighter color along the diagonal top left to bottom right indicate more accurate results. Classification accuracy was highest for blank (100%) and chalk (89.8%), followed by salt (87.5%) and pollen (72.7%), giving a mean accuracy of 87.5%. The dominant error was confusion between pollen and chalk (27.3%). The mean confidence score for each image represents the average probability assigned by the CNN to its predicted class for each sample. For the test data, blank achieved the highest mean confidence (0.988), followed by chalk (0.881), salt (0.851), and pollen (0.711). Note that the confidence reflects the network’s certainty, not correctness, and so misclassifications with high confidence indicate overconfidence. The CNN classification performance on the unseen test set achieved a mean F1-score for all four particle classes (blank, chalk, pollen, and salt) equaling ∼0.87. The relatively low confidence for pollen, combined with its lower recall and F1-score, suggests that this class is more challenging to separate, likely due to overlapping spectral or morphological features with emission events from chalk.

**Figure 4:**
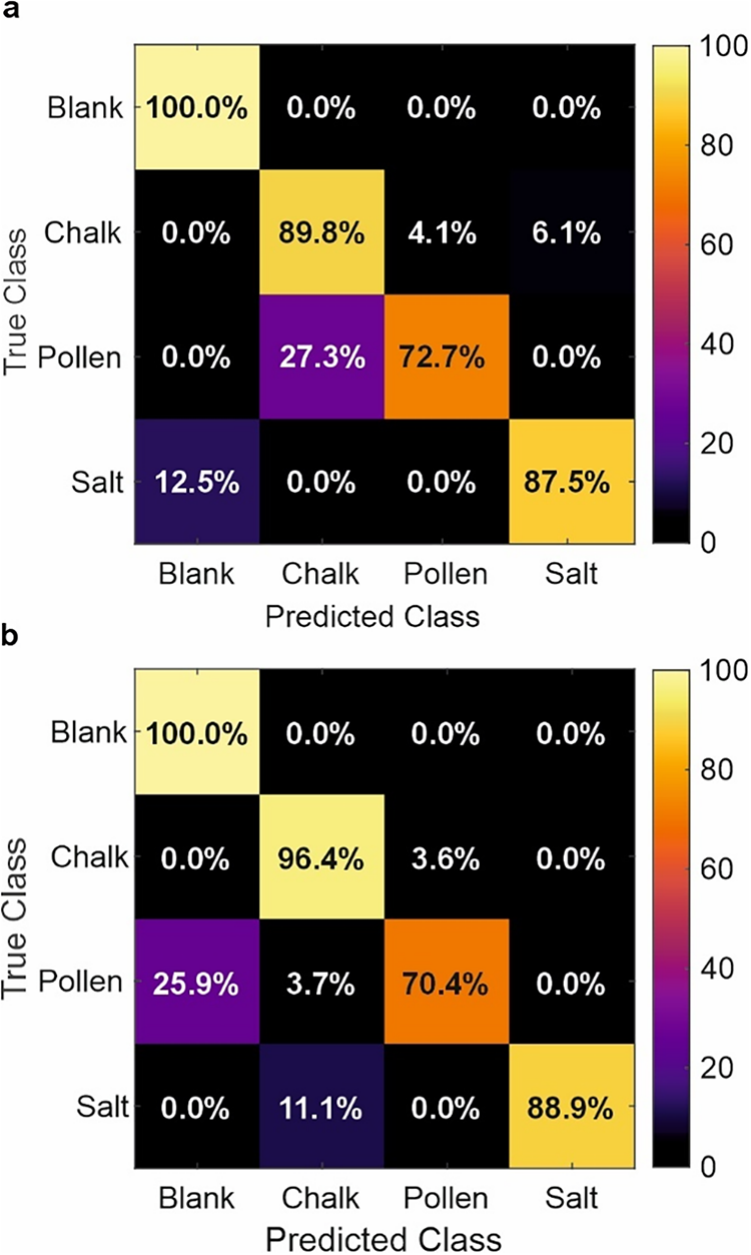
Confusion matrix of the results of classification for the neural network testing on (a) images of optical emission events at 100% laser power and (b) images of optical emission events at 85% laser power.

To test the robustness of the neural network, classification was performed on additional images of emission events taken at 85% full laser power. A total of 107 images were used for testing, split into approximately equal numbers per class (25 blank, 28 chalk, 27 pollen, and 27 salt). The results are displayed in the confusion matrix of Figure 4b, showing that overall performance remained strong, with a mean accuracy of 88.9%. Chalk achieved the highest accuracy (96.4%) and F1-score (0.915), followed by salt (97.2% accuracy and F1-score of 0.941) and blank (93.5% accuracy and F1-score of 0.877). Pollen remained the most challenging class, with an F1-score of 0.809 and classification accuracy of 70.4%, reflecting increased misclassification as blank (25.9%), perhaps due to reduced emission intensity or the size of emission events. The mean confidence scores further illustrate these trends, such that blank and chalk were classified with high certainty (0.990 and 0.956, respectively), salt remained robust at 0.886, while pollen showed the lowest confidence of 0.688, consistent with its reduced accuracy. These results confirm that CNN maintains a strong classification capability under reduced laser power.

To interpret the CNN’s decision-making process, Grad-CAM^38^ was applied to the test data for 100% laser power to generate heatmaps highlighting the image regions most influential for the model’s predictions. We used the second rectified linear unit activation in the third convolutional block for the Grad-CAM extraction, as this midlevel convolutional layer captures fine-grained spatial and spectral features while preserving enough information to interpret the CNN’s focus. Earlier layers tend to encode low-level features such as edges and gradients, while deeper layers abstract semantic content.

Selected correctly classified images are shown in Figure 5a, with their corresponding Grad-CAM visualizations shown in Figure 5b to provide insight into the CNN’s decision-making process. For example, even though there are no particles within the beam for the blank images of the filament, the Grad-CAM activation for the blank filament spans the length of the filament and the background because the network is using a larger region to identify that it is blank, thus indicating that the CNN may be responding to larger structural uniformity rather than smaller discrete particulate features as observed in the other panels for the chalk, pollen and salt classes. Furthermore, classification for chalk and pollen appears to be focusing on small particles and classification for salt appears to focus on broader regions of intensity, such as yellow/orange regions in the image. Intriguingly, in the pollen panel, the CNN selectively highlights green particles while generally ignoring visually prominent pink ones, though both pollen and chalk images contain pink and green particles.

**Figure 5:**
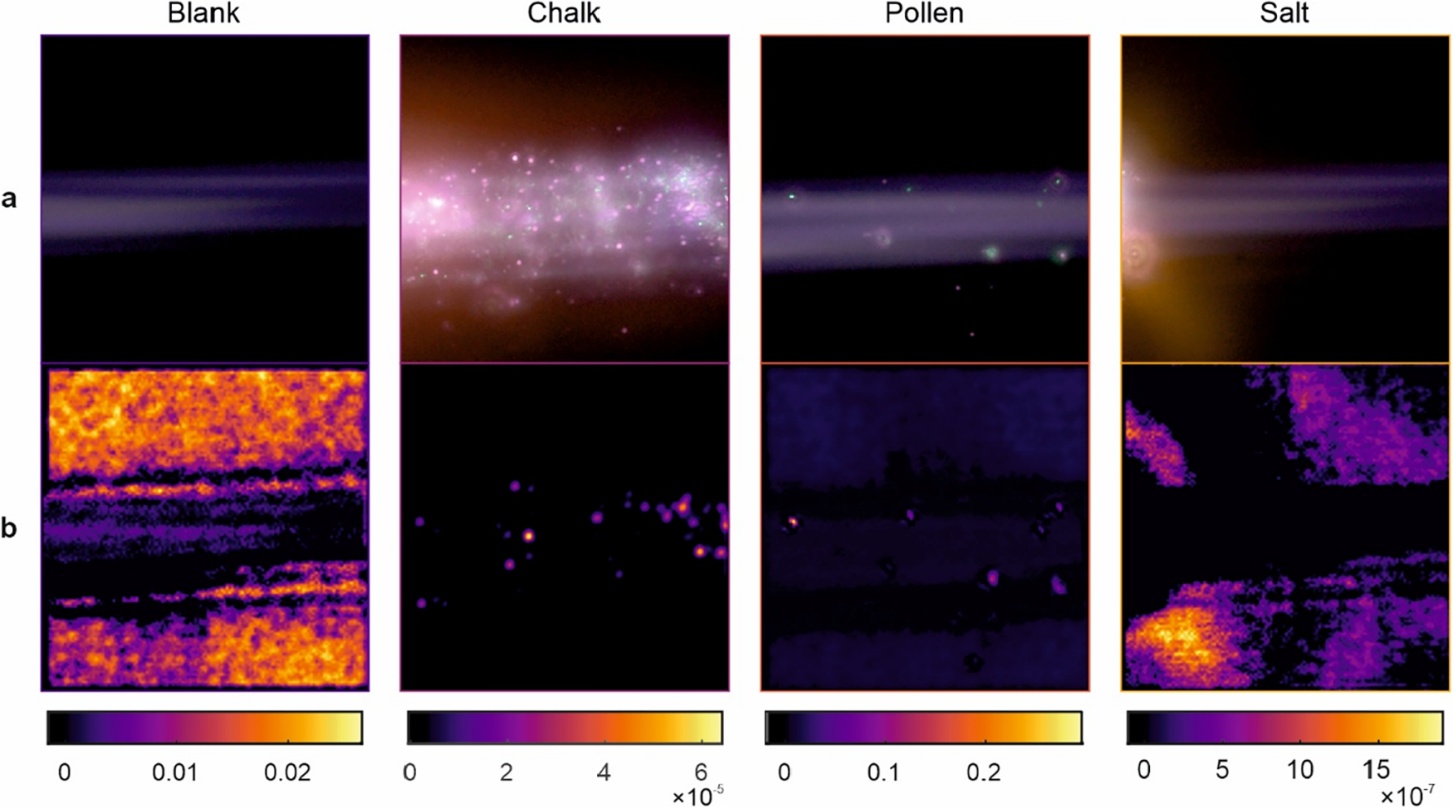
(a) Images of optical emission events for blank (filament only), chalk dust, pollen grains, and salt crystals captured in the filament region and their corresponding Grad-CAM visualizations (the intensity of the brightness of the images has been increased for ease of viewing). (b) Grad-CAM heatmaps highlighting the most influential regions in the CNN model’s prediction.

To investigate the spectral characteristics of particles identified by the CNN, we analyzed normalized chromaticity values (see Figure 6a for red versus green chromaticity plot) extracted from Grad-CAM-highlighted regions (64 × 64 image patches) across the four particle classes on the correctly classified data taken at 100% laser power. From each patch, the top 30 brightest pixels were selected, and chromaticity values were computed for the three most intense Grad-CAM regions per class for correct classification. This approach isolates relative color information rather than raw RGB intensity, reducing dependence on brightness variations across frames.

**Figure 6:**
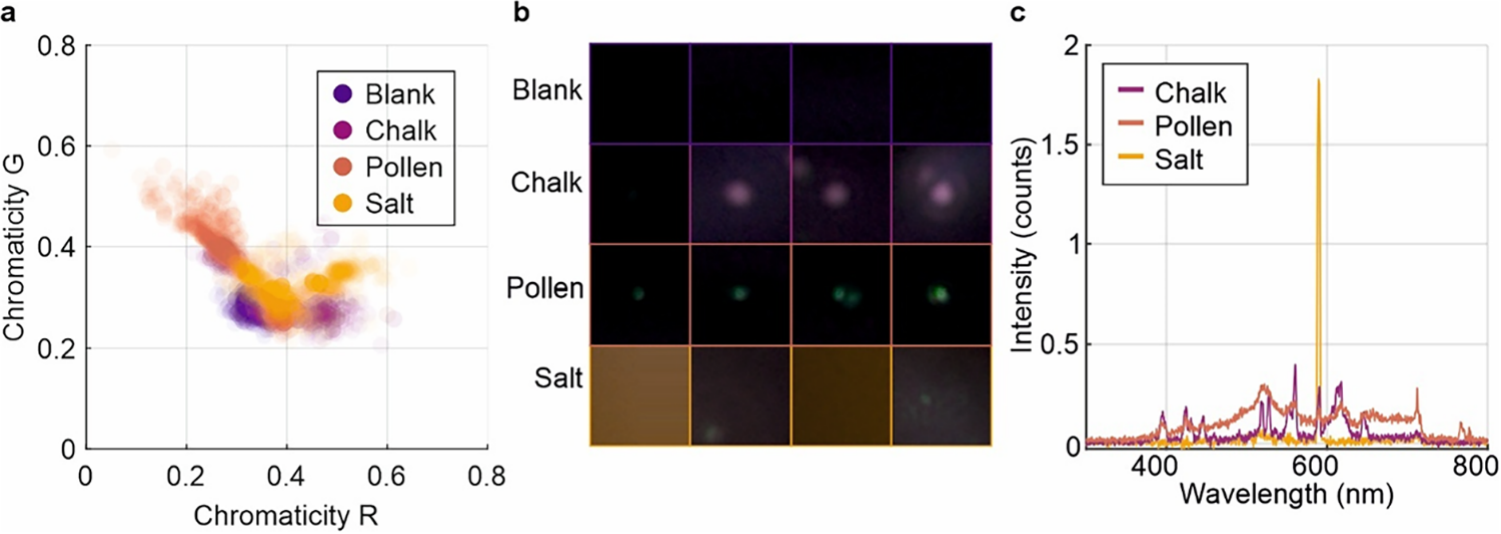
(a) 2D scatter plot of chromaticity values (red versus green) extracted from optical emission events. Each point represents the chromaticity coordinates computed from the top 30 brightest pixels within Grad-CAM-highlighted regions of the original images. Colors indicate particle classes, illustrating the separation and overlap in chromaticity space between blank, chalk, pollen, and salt. (b) Representative Grad-CAM patches for each class, illustrating that chalk often has bright pink features, pollen has distinct green spots, salt has diffuse orange–green patches, and blank frames are dark with minimal signal. (c) The corresponding emission spectra for particles from each class, acquired using 1 s integration times and dark background subtraction.

The scattering plot in Figure 5a reveals partial clustering between classes, and so analysis was used to assess how well particle types separate in color–space. Separation was quantified using the silhouette score, which ranges from −1 to +1, such that values near +1 indicate well-separated clusters, 0 indicates overlap, and negative values suggest misclassification. The weighted mean silhouette score was 0.151 (95% confidence interval of 0.131–0.172), where the confidence interval represents the range within which the true mean is expected to lie with 95% certainty. Blank was strongly distinct (mean = 0.924, median = 0.942, interquartile range (IQR) = 0.038, 100% positive silhouettes, *n* = 1170). The IQR represents the spread of the middle 50% of values, showing that blank’s silhouette scores were tightly clustered. Pollen showed weak but positive structure (mean = 0.081, median = 0.411, IQR = 0.986, 69.5% positive, *n* = 930), indicating a broad distribution with a positive core. On the other hand, chalk (mean = −0.341, 29.0% positive, *n* = 790) and salt (mean = −0.283, 36.5% positive, *n* = 1043) exhibited substantial overlap. Mahalanobis distances (a multivariate measure of separation that accounts for variance and covariance) between chromaticity centroids confirmed chalk and salt as the closest pair (distance = 0.765). These findings indicate that CNN performance relies on combined morphological and spectral cues rather than chromaticity alone.

Grad-CAM visualizations support this interpretation, showing network attention concentrated on localized emission structures, both large and small. These spectral trends can be visually observed in Figure 6b, which presents representative image patches for each particle type. These patches correspond to the actual regions (i.e., image regions in Figure 5a) highlighted by Grad-CAM (the overlays are shown in Figure 5b). Chalk consistently appears as bright, saturated pink spots, pollen as distinct green features, and salt as less intense, diffuse patches exhibiting yellow and a mixture of red and green intensities. Blank regions show little to no signal.

These spectral characteristics are further illustrated in Figure 6c, which shows representative emission spectra for each particle type, acquired using a B&W Tek Glacier X spectrometer during filament interaction at 100% laser power. Each spectrum was recorded with a 1 s integration time and processed by subtracting both the dark background and a filament-only reference to ensure accurate particle-specific emission profiles. It is evident that chalk exhibits multiple sharp peaks across the visible range, consistent with discrete electronic transitions of calcium, which dominate its emission signature.^39^ Pollen exhibits a different profile, characterized by a broad emission band spanning much of the visible spectrum, with a pronounced feature in the green region around 500–550 nm. This behavior is indicative of complex organic fluorophores, such as flavonoids and carotenoids, along with minor contributions from trace metal elements that can introduce weak, narrow emission lines.^40^ This chemical diversity produces a richer spectral signature, aligning with the diffuse green tones observed in Grad-CAM patches and the chromaticity scatter plot. Salt, by contrast, is dominated by an intense feature near 589 nm, corresponding to the sodium D-lines, with only some additional structure in the spectra at other wavelengths.^41^ Collectively, these findings reinforce that particle classification is underpinned by fundamental compositional differences, with chalk and salt presenting highly separable, line-dominated spectra, while pollen occupies a more continuous spectral space that partially overlaps with salt.

The Grad-CAM analysis provides an approximate visualization of regions influencing predictions, but it does not fully capture the network’s full internal feature abstractions. The highlighted regions represent one contributing factor rather than a complete decision basis. Thus, even with overlapping spectral features, the CNN’s ability to achieve high accuracy implies that it also exploits subtle spatial patterns and higher-order feature combinations beyond simple color-space separability.

While in this study the distance between the camera and filament is 18 mm, this work demonstrates a proof-of-principle technique of classifying emission events from a filament. Since filamentation offers a unique advantage for remote aerosol sensing owing to its ability to maintain high peak intensities over extended distances, unlike conventional Gaussian beams that diverge rapidly, this means that strong scattering and ablation signals from particles located tens to hundreds of meters away could be obtained. Although the present study focuses on proof-of-principle measurements under controlled conditions using a side-mounted camera, future methodology could exploit telescopic optics and offset parabolic mirrors for backscatter collection to retrieve signals from any point along the filament, enabling single-ended sensing at long range. Such developments would enable the applicability of filament-based diagnostics from laboratory environments to real-world scenarios, including atmospheric monitoring and remote aerosol characterization.

Although we demonstrate the feasibility of automated particle detection under controlled laboratory conditions, a variety of factors, such as variable airflow and fluctuating humidity and temperature, may influence performance in real-world deployments. Range limitations and weather effects, including condensation or dust accumulation on optical components, may also impact the imaging quality and classification accuracy. To address these constraints, future work will involve systematic augmentation of the image data set under varied environmental conditions and development of an integrated sampling–imaging setup capable of operating in dynamic settings. Iterative refinement of the neural network through feedback from observations across different conditions would improve the detection accuracy and resilience under diverse airflow, humidity, and illumination (sunlight) regimes. Future work will also need to explore adaptive optics and beam shaping to maintain filament stability under the variable conditions present in real-world environments.

The applicability of the proposed method to real-world environments will also require addressing the variety of ambient aerosols present in the atmosphere. For example, ambient aerosols typically comprise a wide variety of complex mixtures with variable morphologies, compositions, and thus optical properties, which can reduce classification confidence and increase the likelihood of interference. While expanding the data set to include a broader range of particle types, alongside the introduction of an ‘unknown’ class and confidence-based thresholds, would further improve robustness, addressing these limitations will likely require alternative neural networks that classify specific regions within an image or scattering event, such as YOLO.^42^ In addition, physics-informed neural networks^43^ offer a promising route to more accurate classification by embedding prior knowledge of laser–matter interaction and characteristic spectral features arising from filamentation-induced ablation. Incorporating these physical constraints within the learning process can guide neural networks toward physically plausible predictions, thereby improving discrimination of complex aerosol mixtures and reducing ambiguity under variable environmental conditions.

## Conclusion

We demonstrated the feasibility of femtosecond laser filamentation combined with optical imaging and lightweight deep learning for remote identification of airborne particulates. Chalk dust, pollen grains, and salt crystals were introduced into the laser filament, where their interaction produced optical emission events that were imaged and subsequently used to train and evaluate a neural network. The model achieved high accuracy across all categories, including when testing emission events occur from a filament producing 85% laser power. A scatter plot of red versus green chromaticity values of optical emission events that were important in the neural network’s classification highlighted blank, chalk, pollen, and salt features having different spectral values. It was therefore found that the neural network attended to discriminative particle features rather than background signal.

While performance may improve with larger data sets and higher frame-rate imaging, this study establishes a clear foundation for real-time remote sensing of single airborne particles. Future work will focus on enhancing robustness by expanding coverage to include a wide range of airborne particles, extending the filament length, and integrating spectral–spatial features to enable multimodal classification.

## Data Availability

The data underlying this study are openly available in the University of Southampton repository at https://doi.org/10.5258/SOTON/D3818.
